# Task demands influence search strategy selection in otoconia-deficient mice

**DOI:** 10.3389/fneur.2025.1531705

**Published:** 2025-04-28

**Authors:** Ryan M. Yoder, Lucas C. Carstensen, Keshav Jagannathan

**Affiliations:** ^1^Department of Psychology, Purdue University Fort Wayne, Fort Wayne, IN, United States; ^2^Department of Mathematics & Statistics, Coastal Carolina University, Conway, SC, United States

**Keywords:** otolith organs, spatial, self-movement, reference memory, Barnes maze

## Abstract

**Introduction:**

The vestibular system plays a crucial role in visual and non-visual navigation. Our recent study found that signals from the otolith organs are necessary for mice’s use of distal visual cues to guide navigation to an invisible goal. Somewhat surprisingly, however, performance was not significantly impaired on some spatial tasks (e.g., Barnes maze reference memory task), questioning the role of otolith signals in visual navigation.

**Methods:**

We report the results of several additional tests of reference memory performance and search strategy use on two versions of the Barnes maze, in an attempt to establish further understanding of the otolithic contribution to visual navigation.

**Results:**

On a small Barnes maze, control mice preferentially used the efficient “spatial” search strategy by the last (8th) day of training, whereas otoconia-deficient *tilted* mice failed to show this preference. On the subsequent probe trial, both groups showed a preference for the former goal location, suggesting otolith signals are not necessary for the use of distal cues to triangulate the animal’s position, relative to distal cues. On a large Barnes maze, both control and *tilted* mice used a spatial search strategy most frequently by the last (4th) day of training and showed a preference for the former goal location on the subsequent probe trial.

**Discussion:**

Overall, these results suggest that otolith dysfunction in mice is associated with subtle navigational deficits that became apparent on the small maze but that were less apparent on the large maze. It is possible that these navigational differences resulted from the greater distance between start and goal locations of the large maze, relative to the small maze. Alternatively, the large maze’s greater distance between the goal and potential alternatives may have facilitated more accurate place recognition.

## Introduction

Efficient navigation typically involves various types of cues, depending on environmental conditions and task demands. Self-movement cues enable the animal to maintain an estimate of its position within novel or non-visual environments, such as darkness (1–4). In contrast, visual environments often contain relatively permanent objects that can serve as landmarks, and most animals learn to use these landmarks to guide their movements (3, 5). However, despite visual landmarks’ dominant control of navigation in familiar visual environments, self-movement cues also contribute to visual navigation in most species, including humans (6) [for review, see (7, 38)]. These self-movement cues arise from several sensory systems, and each provides a unique aspect of the animal’s position or movement within the environment.

Self-movement cues include optic flow, proprioceptive, and vestibular signals. Optic flow occurs when the animal moves relative to fixed external objects, causing the images of these objects to move across the retina (8, 9). Proprioception involves a representation of the motor commands that occur during movements and contributes to gaze stabilization during locomotion (10–12). Signals from the vestibular system represent angular and linear head acceleration, which are detected by the semicircular canals and otolith organs, respectively, and also contribute to gaze stabilization during locomotion (13) [for review, see (14)]. The integrated visual, proprioceptive, and vestibular signals allow the coordination of eye and body movements and facilitate accurate navigational decisions in the presence or absence of familiar landmarks.

We currently have direct evidence that vestibular signals contribute to the neural representations of an animal’s perceived location and directional heading within the environment, which are provided by place cells and head direction cells, respectively. Complete vestibular damage or temporary inactivation disrupts the activity of place cells in the hippocampus, which normally show an increased firing rate when the animal is located in a discrete region of an environment, and head direction cells, which show an increased firing rate when the head is pointed in one direction within an environment (15, 16, 41) [for reviews, see (17, 18)]. This vestibular contribution to place cell activity appears to involve both the semicircular canals and otolith organs [for reviews, see (18–22)]. The directional tuning of head direction cells was lost following inactivation of the semicircular canals, regardless of whether this inactivation included all three canals or was limited to the anterior, posterior, or horizontal canal (23). The head direction signal was similarly disrupted in *epistatic circler* mice that have dysfunctional horizontal canals (24). In otoconia-deficient *tilted* mice, head direction cells were initially detected, but most of these cells lost their directional tuning across trials (25). *Tilted* mice also had place cells that persisted across trials, but their location-specific activity was less robust and they were more likely to represent locations near the arena boundaries, relative to place cells in control mice (26). At this time, no studies have recorded place cell activity following specific elimination of the semicircular canal signals, but the available evidence suggests that signals from both the semicircular canals and otolith organs contribute to place cell and head direction cell activity.

Vestibular signals are necessary for accurate navigational performance in non-visual and visual environments. Intratympanic injection of sodium arsanilate—which destroys the hair cells of the inner ear—impaired rats’ ability to perform a homing task in darkness and impaired the acquisition of a piloting task in light (27, 42). Vestibular dysfunction also caused rats to shift to a response strategy instead of the spatial strategy favored by normal rats (40). This vestibular contribution to navigation appears to include the otolith signals, as *tilted* mice showed more circuitous paths than control mice on a homing task in darkness, but showed less impairment in light (28). On an open-field exploration task, intratympanic sodium arsanilate disrupted the organization of mice’s exploratory movements in both darkness and light, with the greatest impairment in darkness (29). *Tilted* mice also made more circuitous journeys than control mice during non-visual exploration (30). Together, these results suggest the otolith organs contribute more to non-visual navigation than to visual navigation. This is not entirely surprising, but even in a lighted environment, otoconia-deficient *head tilt* mice failed to exceed chance performance on alternation and place recognition tasks on a Y-maze (31). Similarly, *tilted* mice were impaired at using a piloting strategy but were unimpaired at using a beacon strategy, to choose the correct arms on a radial arm maze discrimination task (22). Thus, the otolith organs provide important self-movement cues to support accurate navigation in both non-visual and visual environments, but details of this otolithic contribution to visual navigation are not fully understood at this time.

One way to test the otolithic contribution to navigation is to evaluate the search strategy(ies) used by *tilted* mice to perform a spatial task. This approach is based on several previous studies of mouse navigation that used a reference memory task on a Barnes maze (32). This apparatus is essentially a circular table with numerous holes positioned around the edge, one of which provides escape from an overhead light. This task can be solved with one or more search strategies which have been classified as (1) a “serial” strategy, which involves following the edge of the maze to the goal, (2) a “spatial” strategy, where the animal moves in the direction of the goal, as defined by its position relative to distal cues, or (3) a “mixed” strategy, where the mouse’s trajectory includes aspects of both serial and spatial strategies (33). We tested phenotypically normal and otoconia-deficient *tilted* mice four trials/day, across 4 days, on a small Barnes maze (69 cm diameter). The results indicated that control mice used a serial strategy most frequently at the beginning of training, after which they trended toward a spatial strategy by the last day of training [see Figure 3A in Yoder and Kirby (22)]. In another study, O’Leary and Brown (34) found that normal mice showed a slight preference for a spatial search strategy by the end of training when two training trials occurred per day, across 15 days. Importantly, in both studies, training ended before mice showed a statistically significant preference for the spatial strategy, and it remains unclear whether normal mice would eventually prefer the spatial strategy.

The aforementioned studies evaluated mice’s spatial performance and search strategy use on a small Barnes maze, but mice’s search strategy preference appears to be somewhat different on a larger maze. With the larger (122 cm diameter) maze, mice initially preferred a serial strategy, but this preference decreased across trials as the mice switched to a more efficient spatial strategy (34). We interpret this finding as evidence that the size of the maze or distance to goal can influence which strategy is used to solve the task, with normal mice showing a greater preference for a spatial search strategy on a large maze, relative to a small maze.

In this study, we tested control and *tilted* mice’s performance on a small and large Barnes maze reference memory task. For the small maze, we conducted four acquisition trials/day, across 8 days, to determine whether additional training (relative to our previous study) would prompt control mice to switch to a spatial search strategy. This test also determines whether otolith dysfunction impairs the use of a spatial strategy on this task. For the large maze, we conducted four acquisition trials/day, across 4 days, to determine whether control or *tilted* mice favor a spatial search strategy on the larger maze, where the goal is further from the start location than it is for the smaller maze. On both mazes, we anticipate that control mice, but not *tilted* mice, will favor the spatial strategy during training. We also anticipate that both groups will show a significant preference for the former goal location during the probe trial as they did in our previous study using the small maze (22).

## Materials and methods

### Animals

All procedures involving live animals were approved by the Purdue Animal Care & Use Committee. For Barnes maze testing, otoconia-deficient *tilted* male mice (*n* = 16, aged 4–8 months) and their phenotypically normal heterozygous male littermates (*n* = 16, aged 4–8 months) were obtained from the breeding colony at Purdue University Fort Wayne. The colony was established from an original stock of mice homozygous for the recessive *tilted* mutation (−/−; B6.Cg-Otop1^tlt/J^; Jackson Laboratories, Bar Harbor, ME) that were bred to produce −/− offspring, or crossed with the C57BL/6 J background strain (+/+; Jackson Laboratories) to produce +/− offspring. The resultant −/− and +/− litters were bred to produce +/− and −/− offspring, with a predicted 50% frequency of each genotype in the resulting litter. The +/− descendants were then bred with −/− descendants to maintain the colony in subsequent generations. We chose to use +/− mice as controls, to avoid any behavioral deficits specific to our colony. However, we cannot be certain that our +/− mice performed the same as +/+ mice would perform on the Barnes maze, even though the +/− mice are phenotypically normal and exhibit no known cognitive or motor deficits.

At 12 weeks of age, mice were classified as +/− or −/− using a swim test that has been shown to reliably detect otolith dysfunction in mice (35). This test involves dropping mice from a height of 20 cm into a pool of water. Mice that immediately resurfaced and engaged in normal swimming behavior (an upright posture with the eyes and nose above the surface) were classified as heterozygous controls. Mice that failed to resurface and tumbled underwater were immediately rescued to prevent drowning, and these mice were classified as homozygous *tilted* mice. Mice were between 3 and 8 months of age at the time of testing. We note that some researchers recommend testing for otolith dysfunction with a different procedure, such as a rotarod, that is less stressful than the swim test (31). However, our data suggest the rotarod is not suitable for this purpose, with considerable performance overlap between groups (unpublished observations).

### Apparatus

#### Barnes maze

**Figure 1:**
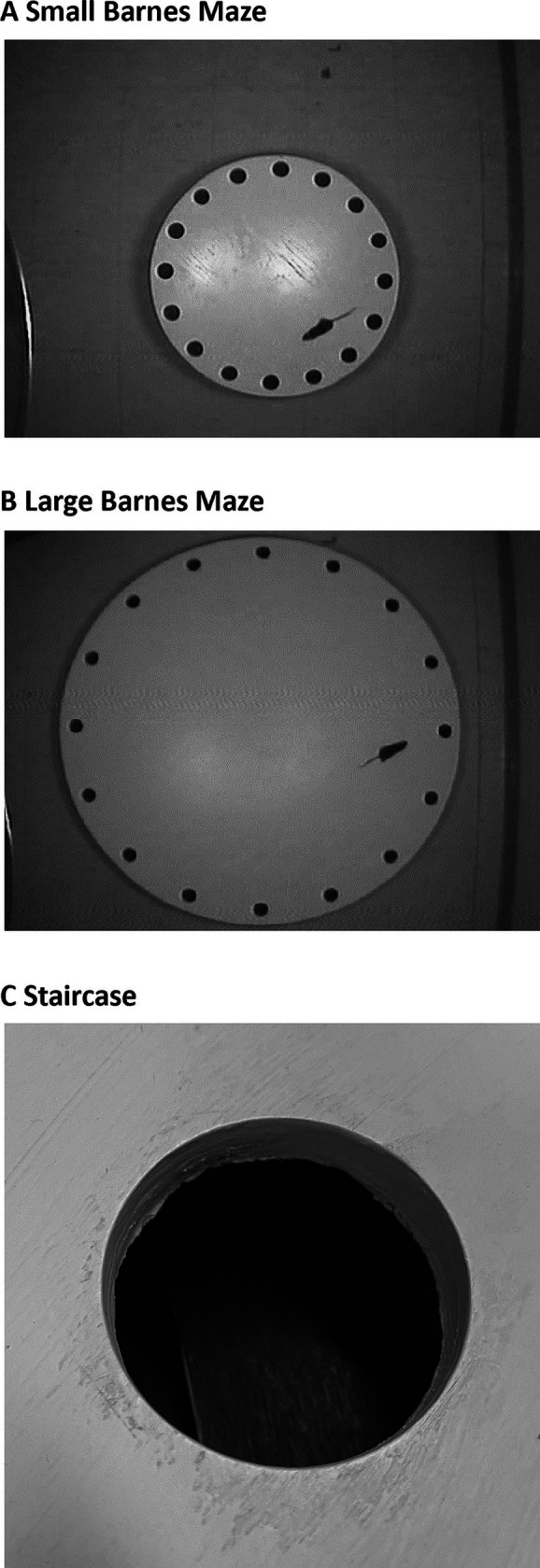
Small and large Barnes mazes. Mice were tested on a small Barnes maze (**A**; 69 cm diameter) and large Barnes maze (**B**; 120 cm diameter) in the same physical location, but on different days. **(C)** Close-up view of the staircase leading into the escape box.

Two versions of the Barnes maze were used. The small Barnes maze (Figure 1A) was constructed from a circular piece of plywood (69 cm diameter), painted white, with 16 circular holes (diameter = 4.5 cm) located along the edge, as described previously (22). The large Barnes maze (Figure 1B) was constructed in a similar manner but had a larger diameter overall (120 cm); angular distance between the 16 holes remained the same as for the small maze, but linear distance between holes increased in proportion to the maze diameter. For both mazes, a black wooden escape box could be mounted under one of four holes. A plywood subfloor, painted black, prevented entry to the non-goal escape holes. Two downward-facing 150-watt incandescent light bulbs mounted overhead in aluminum reflectors served as an aversive stimulus, and all other room lights were extinguished. The overhead lights illuminated the maze surface, room walls, and various extramaze objects (e.g., desk and cabinet) that could serve as spatial cues. An overhead video camera was used to record the position of the mouse during maze performance at 30 fps. Both mazes were located in the same position in the testing room (on different days), ensuring that the same constellation of distal room cues was available for both mazes.

### Procedure

#### Habituation

Each mouse had one 5-min habituation trial to familiarize it with the maze and the escape box. The overhead lights were turned on, and the mouse was confined to a transparent cylinder around the area including the target escape hole and during confinement, the mouse was allowed to enter the escape hole and explore the immediate area. If the mouse did not enter the escape hole within 5 min, the mouse was guided toward the escape hole. One *tilted* mouse did not voluntarily enter the escape box, and it was therefore placed in the box for 30 s before being returned to the home cage; all others voluntarily entered the hole within the 5-min habituation trial.

An important consideration is whether *tilted* mice are capable of the motor functions necessary to complete the Barnes maze task (i.e., walking and climbing into the goal box). We did not explicitly test the motor skills of the mice used here, but previous studies have clearly demonstrated that otoconia-deficient mice are capable of walking at approximately the same speed as controls in various tasks, albeit with an abnormal head posture (22, 28, 30, 31, 35). In contrast, otoconia-deficient mice maintain an abnormal posture while walking across narrow surfaces (45) and may, therefore, be unable to climb as well as control mice. Accordingly, our initial efforts to use a Barnes maze with *tilted* mice suggested that these animals had some difficulty or reluctance to enter the escape hole (unpublished data). To address this issue, we constructed a wooden staircase, painted black, that led from the table top into the escape box (Figure 1C). The top stair was approximately 2 cm below the table surface and was, therefore, not visible to a mouse until it came within several cm of the hole.

#### Acquisition and probe trials

Mice were tested in squads of four, with two heterozygous controls and two homozygous *tilted* mice per squad. Hole assignment was counterbalanced across squads. The maze was cleaned between trials to remove potential odor cues, and the maze was rotated 90° each day.

Video recording started before the mouse was released from the transparent cylinder and stopped after all four paws entered the escape hole. Four acquisition trials were conducted per day, across 8 days for the small maze and 4 days for the large maze. Mice were first restricted to the center of the maze for 15 s by a transparent cylinder, after which the cylinder was removed and the mouse was free to explore and search for the escape hole. The trial started when the cylinder was removed and ended when the mouse placed all four paws in the escape hole. Mice that did not enter the escape hole after 5 min were guided to the escape hole by the experimenter. The mouse then remained in the escape box for 30 s before returning to the home cage and then remained in the home cage for at least 10 min between trials. A single 5-min probe trial was conducted 1 day after the last acquisition trial with no escape available.

### Scoring and analysis

Performance measures for acquisition trials included running speed during the initial phase of the search (from the point of release until the first nose poke into a hole or arrival at the edge of the table, whichever came first), latency and distance from the start location to the escape hole, and the frequency of errors that occurred before entering the escape hole. An error occurred when a mouse poked its head into a hole that did not lead to the escape box. Consecutive nose pokes into the same hole were counted as one error unless these pokes were separated by a nose poke into a different hole. For all measures, performance scores for the four daily trials were averaged for each animal, and daily averages were calculated within groups. Group scores were compared with a mixed Day X Group analysis of variance (ANOVA), and a Huynh-Feldt correction was used for Sphericity violations.

Search strategy was categorized as *serial*, *spatial*, or *mixed*, as described previously (22, 32, 33). A mouse uses a serial strategy when it walks to the edge of the maze and follows the edge and/or false holes until it reaches the goal. A search path was spatial if a mouse moved in the direction of the escape hole and did not explore a false hole located more than two holes away from the correct hole. This area corresponded to a quadrant of the maze. A mixed strategy includes aspects of both serial and spatial strategies or otherwise did not meet the criteria for either of these classifications. Chi-square tests were used to compare search strategy use within and between groups.

For the probe trial, each mouse explored the maze for 5 min, but only the first 3 min (180 s) were used for analysis. This time point was chosen because a previous report indicated that mice tend to explore non-goal areas after approximately 3.5 min of a probe trial (33). The maze was divided into quadrants: the *goal* quadrant contained the former goal location, the two adjacent quadrants were labeled as *right* and *left* (as viewed from the center of the maze), and the *opposite* quadrant was located across from the goal quadrant. The mean time spent in all quadrants is presented, and time in each quadrant was compared between groups.

## Results

No mice were excluded from the acquisition analysis, but some *tilted* remained near the escape hole for extended periods of time during the first several trials on both mazes. This pattern suggests they recognized the goal location but either had difficulty entering the escape hole or were reluctant to enter. However, all mice voluntarily entered the escape hole by the third training trial. Several *tilted* mice also walked with splayed limbs, but their walking speed did not differ from mice that walked with a normal posture (discussed below).

### Small Barnes maze acquisition

**Figure 2:**
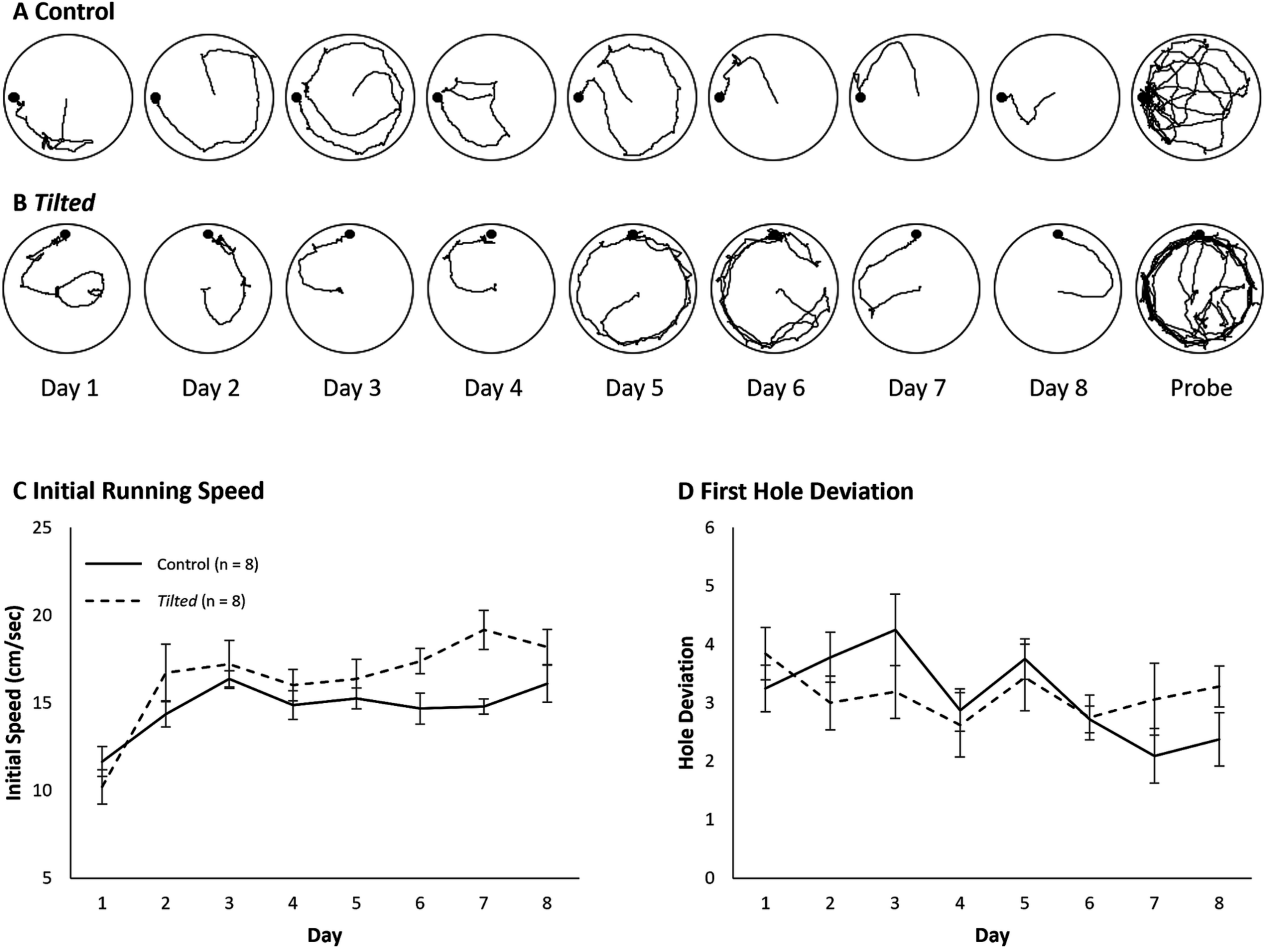
Example search paths, running speed, and first hole deviation on the small Barnes maze. **(A,B)** Example search paths from one control and one *tilted* mouse, for training days and the probe trial. **(C)** Running speed during the initial phase of the task was similar between groups, although this comparison approached significance. Running speed increased across days for both groups, and the interaction was significant. **(D)** Deviation between the goal and first hole visit is shown for each day of training. Hole deviation decreased significantly across trials, but the group and interaction effects were not significant. Mean ± SEM.

Example paths for one control and one *tilted* mouse are shown for all days of testing (Figures 2A,B). The initial phase of the search typically involves movement from the center toward the edge, and we evaluated running speed during this phase to verify that all mice were able to navigate the maze (Figure 2C). Mean daily speed was compared between groups and across days with a Day X Group mixed ANOVA. Control mice’s speed was not significantly different from that of *tilted* mice, although this comparison approached significance (F(1, 14) = 3.49, *p* = 0.083, η^2^_p_ = 0.200). However, overall speed increased significantly across days (F(7, 98) = 12.57, *p* < 0.001, η^2^_p_ = 0.473), and the interaction was significant (F(7, 98) = 2.271, *p* = 0.035, η^2^_p_ = 0.140). Thus, both groups increased their initial movement speed as training trials progressed, and the *tilted* mice trended toward greater initial speed than control mice. This trend was somewhat surprising, given that several *tilted* mice exhibited splayed limbs during immobility, but this postural abnormality did not reduce running speed during locomotion.

We then quantified the absolute deviation between the goal and the first hole visited for each trial, as a measure of navigation accuracy (Figure 2D). The mean absolute deviation was calculated for each animal for each day and then averaged within groups. A Day X Group mixed ANOVA revealed that deviation decreased across trials overall (F(7, 98) = 2.525, *p* = 0.02, η^2^_p_ = 0.153), but the group and interaction effects were not significant (*p* > 0.05). Thus, both groups’ accuracy improved across training trials.

**Figure 3:**
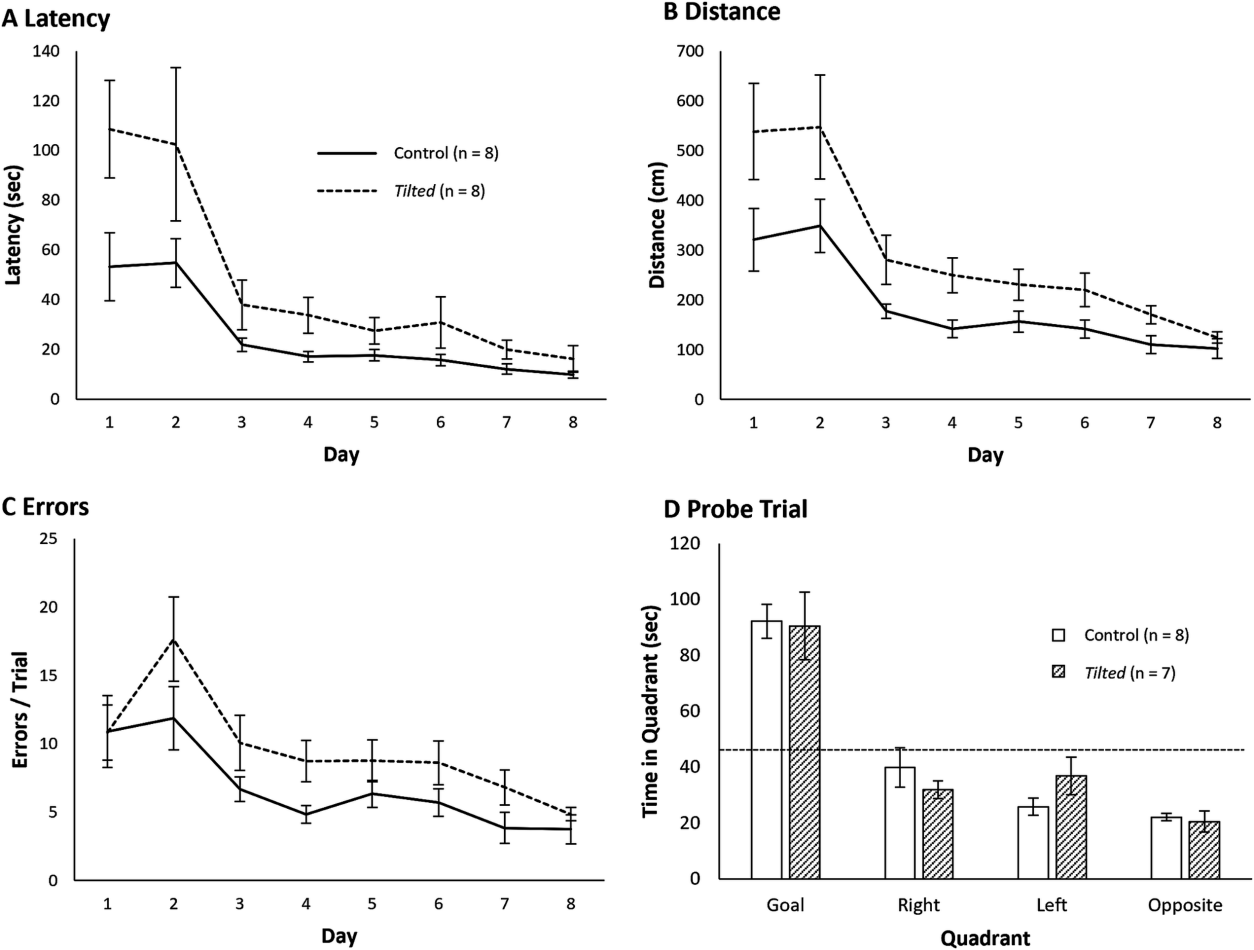
Performance on the small Barnes maze. Control and *tilted* mice showed significant group differences in latency to reach the goal **(A)**, distance to goal **(B)**, and error rates **(C)** but showed similar performance improvements across days. **(D)** On the subsequent probe trial, time spent in the former goal quadrant was significantly greater than expected by chance (45 s; dotted line). Groups did not differ in time spent within any quadrant. Mean ± SEM.

Mean latency, distance, and error scores are presented in Figures 3A–C, respectively, and statistical comparisons are presented in Table 1. For all three measures, *tilted* mice’s scores showed considerably more variability during the first several days, relative to those of control mice. A separate Day X Group mixed ANOVA for each measure revealed a significant main effect of group, with control mice showing shorter latency to reach the goal, shorter distance traveled, and fewer errors, relative to *tilted* mice. These comparisons also revealed significant main effects of day, but none of the comparisons revealed a significant interaction. Together, these results suggest that control mice performed better than *tilted* mice overall, but both groups showed performance improvements across 8 days of acquisition training on the small Barnes maze.

**Table 1:** Group comparisons of latency, distance, and error rates during acquisition training on the small Barnes maze.

Latency	df	F	*p*	η^2^_p_
Group	1, 14	6.30	0.025	0.310
Day	2.46, 34.4	15.30	< 0.01	0.522
Day × Group	2.46, 34.4	1.80	0.173	0.114
Distance				
Group	1, 14	14.31	0.002	0.505
Day	3.4, 48.1	15.70	< 0.01	0.528
Day × Group	3.4, 48.1	1.10	0.360	0.073
Errors				
Group	1, 14	6.89	0.020	0.330
Day	5.28, 73.9	8.67	< 0.01	0.382
Day × Group	5.28, 73.9	0.61	0.700	0.042

Huynh-Feldt correction was applied for Sphericity violations.

### Small Barnes maze probe trials

One *tilted* mouse was removed from the analysis because it fell from the maze before reaching 180 s. Mean time spent in each quadrant of the maze is shown in Figure 3D. Both control mice (M = 92.2 ± 6.08 s) and *tilted* mice (M = 90.6 ± 12.07 s) spent the greatest amount of time in the former goal quadrant; time in the goal quadrant was significantly greater than expected by chance (45 s) for control (t(7) = 7.77, *p* < 0.001, d = 2.75), and *tilted* mice (t(6) = 3.78, *p* = 0.009, d = 1.43). In addition, separate independent-groups *t*-tests showed that time spent in each of the quadrants was similar between groups (all *p*’s > 0.05). Thus, both control and *tilted* mice appeared to accurately recall the goal location, relative to distal room cues, and spent approximately half of the probe trial in the former goal quadrant.

### Small Barnes maze search strategy analysis

**Figure 4:**
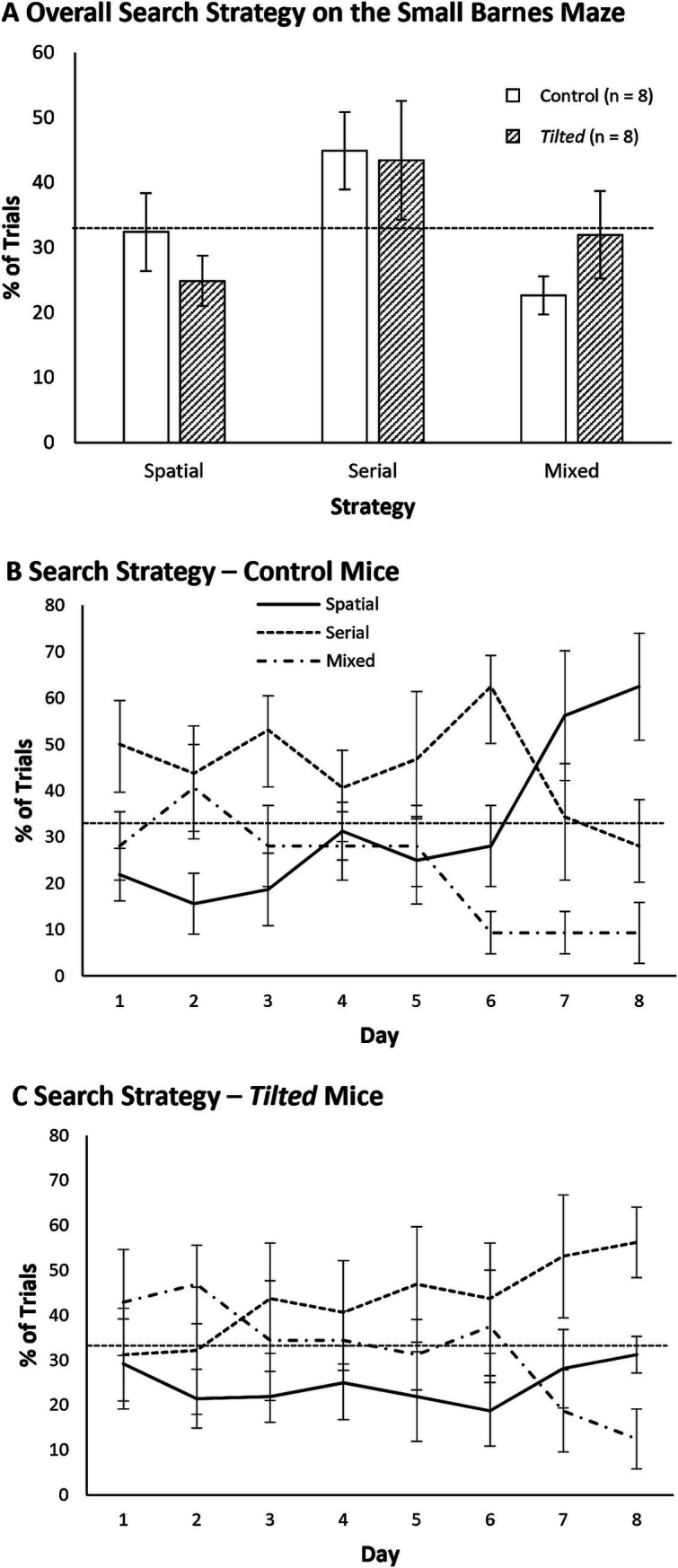
Search strategy on the small Barnes maze. **(A)** The serial strategy was most common across all training trials for control and *tilted* mice. **(B,C)** Control mice increased the use of a spatial strategy across training trials, and *tilted* mice increased the use of a serial strategy. On the last 2 days of training, control mice used a spatial strategy more often than expected by chance, whereas *tilted* mice used a serial strategy more often than expected by chance. Dotted line indicates chance expectation (33%). Mean ± SEM.

We initially collapsed the frequency for each search strategy across all training trials and then compared these frequencies between groups with separate *t*-tests; Figure 4A shows these data as percentages. Both groups showed similar overall frequencies of spatial, serial, and mixed strategies (all *p*’s > 0.05).

We then evaluated search strategy each day, to determine whether either group switched strategies across training days. Figures 4B,C present percentages of all three strategies for all training days. A chi-square test for independence revealed that the frequencies of spatial, serial, and mixed strategies were different between training days 1 and 8 for control mice (χ^2^ (2, *N* = 64) = 11.22, *p* < 0.01) and *tilted* mice (χ^2^ (2, *N* = 64) = 6.68, *p* < 0.05). Furthermore, the frequencies of the three strategies were different between groups on day 8 (χ^2^ (2, *N* = 64) = 6.48, *p* < 0.05), with control mice using the spatial strategy most frequently and *tilted* mice using the serial strategy most frequently. Thus, control mice appeared to switch to the more efficient spatial search strategy by the end of training on the small maze, whereas *tilted* mice favored the less efficient serial strategy at the end of training.

### Large Barnes maze acquisition

**Figure 5:**
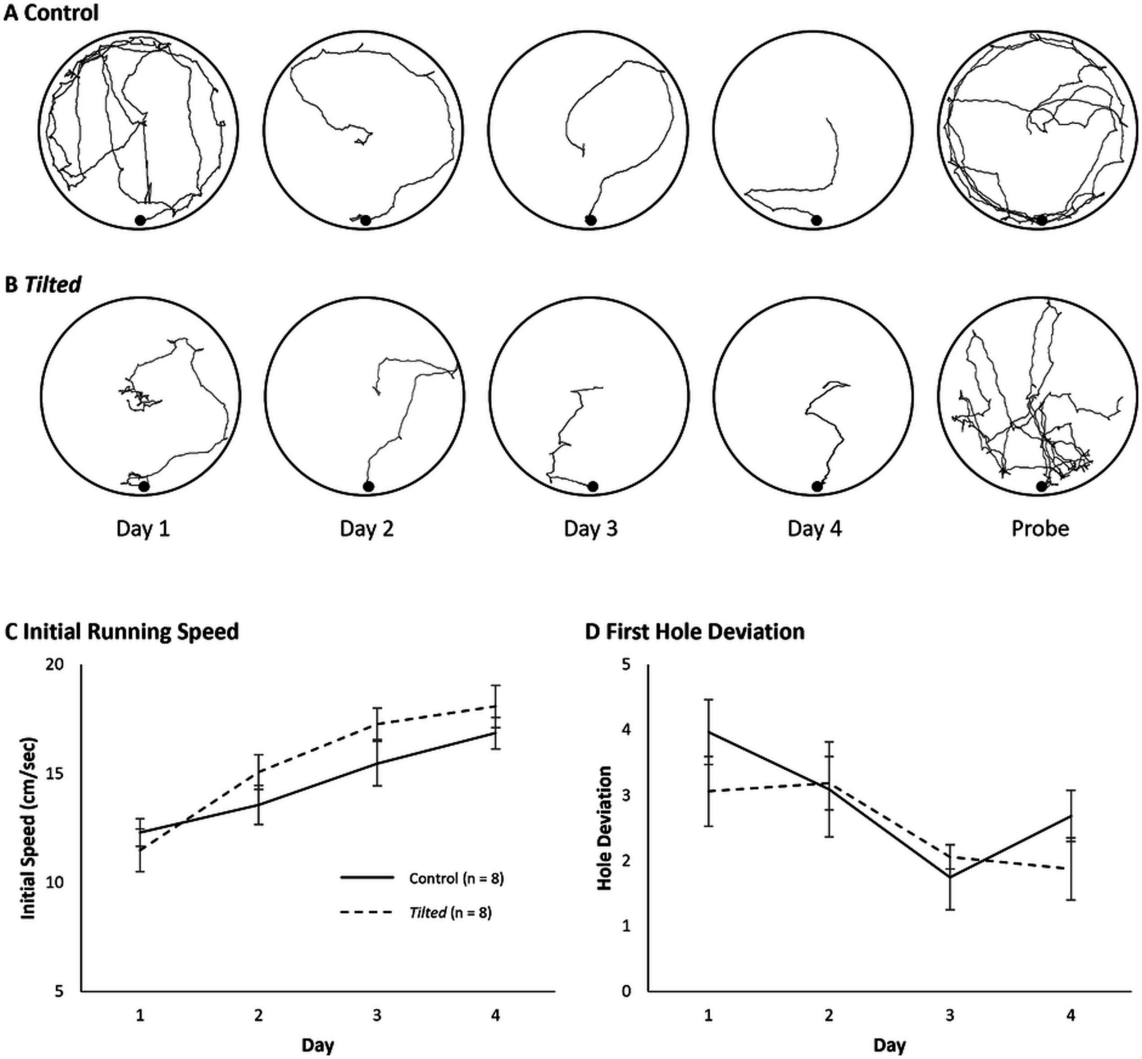
Example search paths, initial running speed, and first hole deviation on the large Barnes maze. **(A,B)** Example search paths from one control and one *tilted* mouse, across all training days and the probe trial. **(C)** Running speed during the initial phase of the task was similar between groups but increased across days for both groups, and the interaction was significant. **(D)** Absolute deviation between the goal and first hole visited is shown for each day of training. Deviation scores decreased significantly across trials, but group and interaction effects were not significant. Mean ± SEM.

Example search paths are shown for acquisition and probe trials in Figures 5A,B. We first evaluated mean daily running speed during the initial phase of the task and compared these speeds between groups and across days with a mixed Day X Group ANOVA (Figure 5C). Control mice’s speed was not significantly different from that of *tilted* mice overall (F(1, 14) = 0.761, *p* = 0.398, η^2^_p_ = 0.052). However, overall speed increased significantly across days (F(3, 42) = 58.4, *p* < 0.001, η^2^_p_ = 0.807), and the interaction was significant (F(3, 42) = 3.483, *p* = 0.024, η^2^_p_ = 0.199). Thus, both groups increased their initial movement speed as training trials progressed, and the increase was somewhat greater for *tilted* mice than for control mice.

We then quantified the absolute deviation between the goal and the first hole visited for each trial, as a measure of navigation accuracy, as described above for the small maze (Figure 5D). A Day X Group mixed ANOVA revealed that deviation decreased across trials overall (F(3, 42) = 7.523, *p* < 0.001, η^2^_p_ = 0.350), but the group and interaction effects were not significant (*p* > 0.05). Thus, both groups’ accuracy improved across training trials on the large maze.

**Figure 6:**
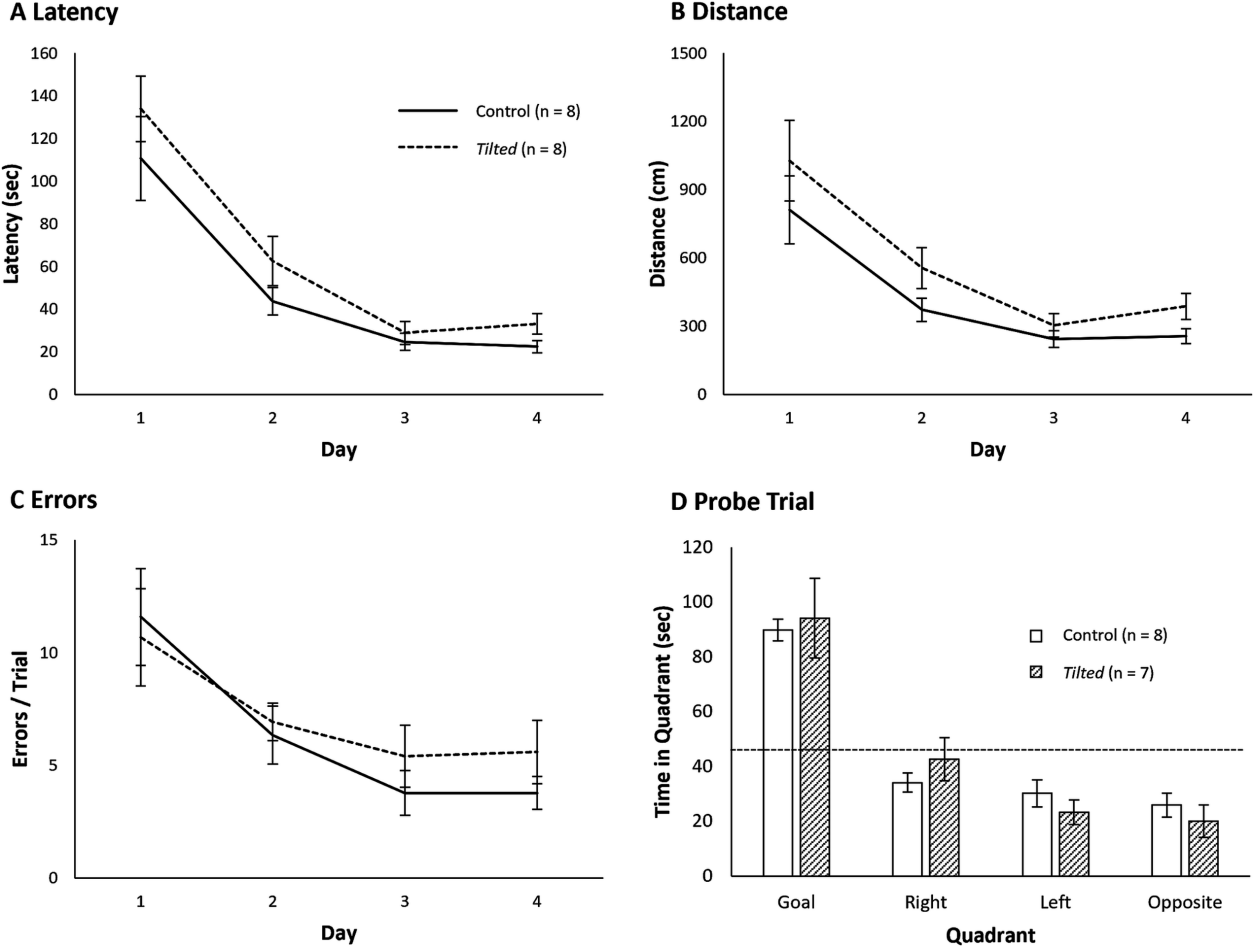
Performance on the large Barnes maze. Control and *tilted* mice showed similar latency to reach the goal **(A)**, distance to goal **(B)**, and error rates **(C)**, and performance improved at similar rates across days. **(D)** On the subsequent probe trial, time spent in the former goal quadrant was significantly greater than expected by chance (45 s; dotted line). Time spent in any quadrant was similar between groups. Mean ± SEM.

Mean latency, distance, and error scores are presented in Figures 6A–C, respectively, and statistical comparisons are presented in Table 2. A separate Day X Group mixed ANOVA for each measure failed to reveal a significant main effect of group; control and *tilted* mice showed similar latency to reach the goal, distance traveled, and errors rates. All three comparisons revealed significant main effects of acquisition day (*p* < 0.05), but none of the interactions were significant (*p* > 0.05). Together, these results suggest that otolith dysfunction did not impair mice’s ability to learn the location of the escape hole across 4 days of acquisition training on the large Barnes maze.

**Table 2:** Group comparisons of latency, distance, and error rates during acquisition training on the large Barnes maze.

Latency	df	F	*p*	η^2^_p_
Group	1, 14	3.73	0.074	0.210
Day	1.74, 24.34	37.05	< 0.01	0.726
Day × Group	1.74, 24.34	0.33	0.690	0.023
Distance				
Group	1, 14	3.68	0.076	0.208
Day	1.65, 23.09	21.45	< 0.01	0.605
Day × Group	1.65, 23.09	0.29	0.710	0.020
Errors				
Group	1, 14	0.49	0.500	0.034
Day	2.20, 30.73	9.31	< 0.01	0.400
Day × Group	2.20, 30.73	0.39	0.700	0.027

Huynh-Feldt correction was applied for Sphericity violations.

### Large Barnes maze probe trials

One *tilted* mouse fell from the maze prior to reaching 180 s, and this animal was removed from the analysis. Mean time spent in each quadrant of the maze is shown in Figure 6D. Control (M = 89.9 ± 3.92 s) and *tilted* mice (M = 94.1 ± 14.50 s) spent the greatest amount of time in the former goal quadrant, relative to in any other quadrant. Time in the goal quadrant was significantly greater than expected by chance (45 s) for control (t(7) = 11.43, *p* < 0.001, d = 4.04) and *tilted* mice (t(6) = 3.38, *p* = 0.015, d = 1.28). Separate independent-groups *t*-tests revealed that time in each of the quadrants did not differ between groups (all *p*’s > 0.05). Thus, as with the small maze, both control and *tilted* mice appeared to accurately recall the former goal location on the large maze, relative to distal room cues.

### Large Barnes maze search strategy analysis

**Figure 7:**
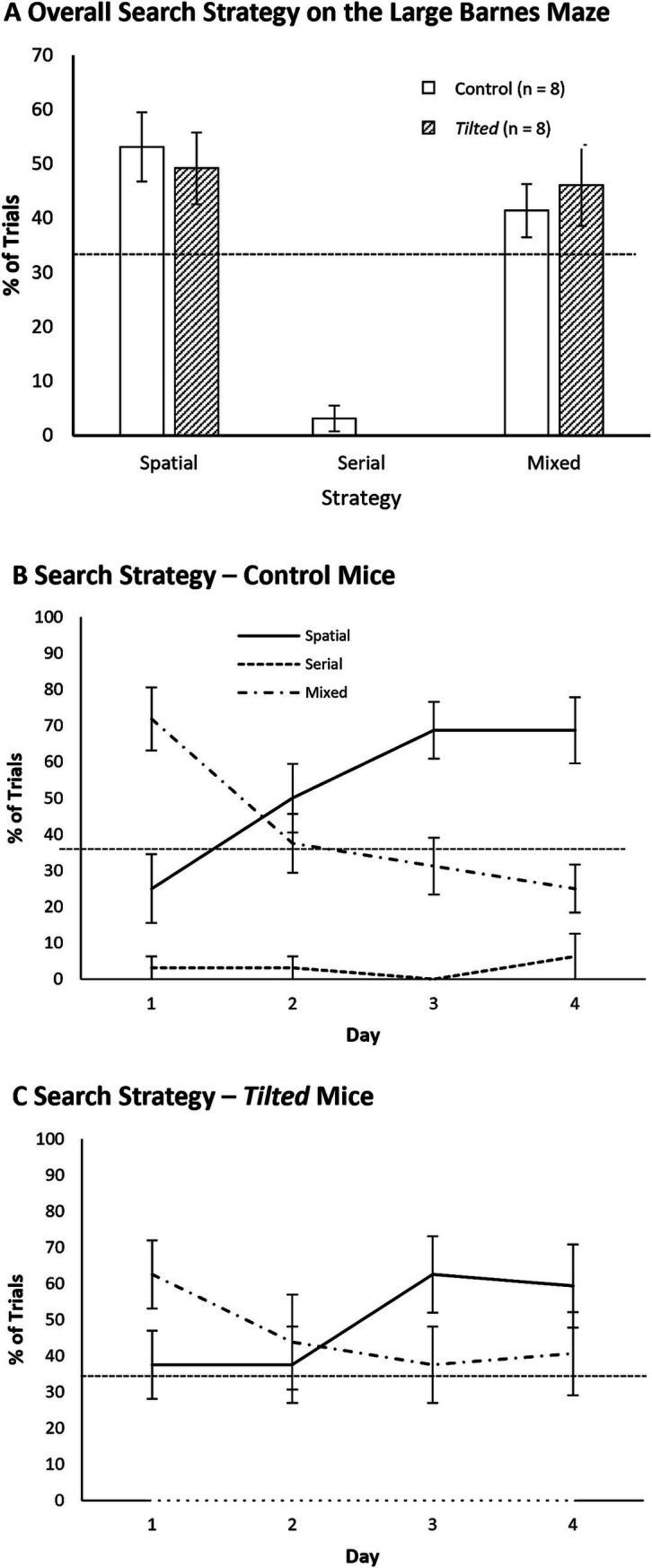
Search strategy on the large Barnes maze. **(A)** The spatial strategy was most common overall, but both groups used the mixed strategy nearly as often; the serial strategy was rarely used. **(B,C)** Both groups used the mixed strategy more than expected by chance at the beginning of training but then favored the efficient spatial strategy during the last 2 days. Dotted line indicates chance expectation (33%). Mean ± SEM.

We first collapsed the frequency of each search strategy across all trials and compared these overall frequencies between groups with separate *t*-tests (Figure 7A shows these data as percentages). Both groups showed similar use of spatial, serial, and mixed strategies (all *p*’s > 0.05). We then evaluated search strategy between days 1 and 4, to determine whether either group switched strategies across training days. Figures 7B,C show percentages of all three strategies as a function of acquisition day. The frequencies of spatial, serial, and mixed strategies were significantly different between training days 1 and 4 for control mice (χ^2^ (2, *N* = 64) = 14.13, *p* < 0.01) but not for *tilted* mice (χ^2^ (2, *N* = 64) = 3.07, *p* > 0.05). We then compared the groups’ strategy within the last day of training. Strategy selection was not different between groups on day 4 (χ^2^ (2, *N* = 64) = 3.41, *p* > 0.05), with both control and *tilted* using the spatial strategy most frequently. Thus, both control and *tilted* mice appeared to use the more efficient spatial search strategy on the large Barnes maze, although *tilted* mice used the serial strategy nearly as often as the spatial strategy on the last day. Interestingly, both groups rarely used the mixed strategy on the large maze.

### Comparison of search strategies on day 4 of small and large mazes

This study was not initially designed as a direct comparison of navigational performance between small and large mazes, but the difference in strategy at the same point in training (Day 4) provides insight into control and *tilted* mice’s spatial performance. On the small maze, control and *tilted* mice showed similar frequencies of strategy selection on day 4 (χ^2^ (2, *N* = 64) = 0.42, *p* > 0.05), with both groups using the serial strategy most often (see Figures 4B–C). On the large maze, groups also showed similar strategy selection, but here, both groups used the spatial strategy most often (see Figures 7B–C). We then compared each group’s strategy selection on day 4, between the two mazes. Strategy selection was significantly different for both groups on the small maze, versus the large maze: control (χ^2^ (2, *N* = 64) = 12.63, *p* < 0.01); *tilted* (χ^2^ (2, *N* = 64) = 17.65, *p* < 0.01). Overall, both control and *tilted* mice used a serial strategy most often on day 4 on the small maze but used a spatial strategy most often on day 4 of the large maze. This result suggests the large maze’s greater size, distance to goal, or linear distance between holes encouraged control mice to switch to a spatial strategy at an earlier time point than they did on the small maze. The large maze also encouraged *tilted* mice to preferentially use a spatial strategy by the end of training, whereas they never switched to a spatial strategy on the small maze, even after 8 days of training.

## Discussion

We evaluated the navigational performance and search strategy use in phenotypically normal control and otoconia-deficient *tilted* mice on a small Barnes maze across 8 days of acquisition training followed by a single probe trial and on a large Barnes maze across 4 days of acquisition training followed by a single probe trial. On the small Barnes maze, both groups showed performance improvements across days, but only the control mice showed a significant preference for the efficient spatial strategy by the end of acquisition training. However, both groups were able to distinguish the former goal location on the subsequent probe trial. On the large Barnes maze, where animals were required to walk a greater distance between the start and goal locations, both groups showed performance improvements across days and used a spatial search strategy most frequently by the end of acquisition training. Both groups were also able to distinguish the former goal location on the subsequent probe trial. These results are consistent with previous demonstrations of an otolithic contribution to navigational performance, but the distance required to reach the goal, and/or the linear distance between the goal and other holes, may determine the extent to which otolith dysfunction affects the strategy used for navigation.

Efficient navigation requires the ability to triangulate one’s orientation and position, relative to distal landmarks. Signals from the otolith organs contribute to the stability of an ongoing representation of head/body orientation, as indicated by degraded head direction signal in *tilted* mice (25). Accordingly, signals from the otolith organs are also necessary for the use of a piloting strategy to solve a discrimination task on a radial arm maze, where the directional choice must be made at the start of the trajectory (22). However, otolith signals do not appear to contribute exclusively to the animals’ perceived orientation but also appear to contribute to the ability to perceive one’s location. Hippocampal place cells reliably exhibit a higher firing rate when phenotypically normal mice entered a small region of an environment, whereas *tilted* mice had larger and less precise place cell representations (26). *Tilted* mice’s place fields were also more likely to occur near a tactile cue (wall) than in control mice, suggesting they were mildly impaired at using distal cues to triangulate their location. We interpret these findings as evidence for strong otolithic involvement in orientation perception and somewhat lesser involvement in place recognition. Because both of these representations are necessary for the use of a spatial search strategy, the present *tilted* mice’s failure to reliably switch to the spatial strategy on the small maze is not entirely surprising. However, an alternative explanation is that the *tilted* mice have motor deficits that impair their ability to move efficiently about the maze. If this was the case, however, we would expect their average speed to be lower than that of controls. Because we did not see this speed reduction, we do not think the observed group differences resulted from motor deficits in the *tilted* mice.

The large Barnes maze was designed to increase the distance to the goal and the linear distance between holes, relative to the small Barnes maze. Normal mice are known to favor the use of visuo-spatial cues when solving the large Barnes maze (33), and we interpret this as evidence that the large maze is better suited than the small Barnes maze to evaluate spatial performance in mice. Accordingly, our control mice preferentially used a spatial search strategy by the fourth day of training on the large maze, whereas this preference did not appear until later on the small maze. Interestingly, however, the *tilted* mice also used a spatial search strategy most frequently at the end of training on the large maze, suggesting they were not impaired at using visuo-spatial cues for navigation on this task. This finding was somewhat surprising, given that *tilted* mice were impaired at using a spatial strategy to choose the correct arms on a radial arm maze discrimination task (22). One possibility is that the larger maze allows the animal to correct its trajectory between the start and goal location, before it makes an error(s). However, even the control mice showed somewhat circuitous and variable trajectories, often visiting one or two incorrect hole(s) even if they were using a spatial strategy. Therefore, we do not think path circuity explains the different strategy selection between the large and small mazes. Another possibility is that the greater linear distance between the goal and the adjacent false holes on the large maze, relative to the small maze, enabled more accurate discrimination of the goal location. This interpretation is consistent with our previous demonstration that *tilted* mice’s place fields are less precise than those of control mice (26). However, we are not aware of any previous efforts to quantify the limits of place recognition in normal or *tilted* mice.

### Relevance to human navigation and vestibular dysfunction

The development of technology, specifically virtual reality (VR), offers another approach to the study of spatial functions in animals and humans experiencing vestibular dysfunction. Early VR studies showed that the hippocampus and related brain regions become highly active during virtual navigation, as with real-world navigation (36). However, the apparatus used for VR studies requires the individual to remain motionless, thus eliminating the head movement and vestibular activation associated with real-world movements [for review, see (22, 37)]. The present demonstration of subtle deficits associated with otolith dysfunction, along with our previous demonstrations of otolithic contributions to select aspects of navigation, suggests that otolith dysfunction may manifest differently in VR navigation than in real-world navigation. However, even if VR navigation does not require the same vestibular input as real-world navigation, patients with vestibular dysfunction show deficits on VR navigation tasks (43, 44). The impaired VR navigation in vestibular patients suggests a necessary vestibular contribution to VR navigation, even though the navigator lies motionless. Additional studies of vestibular signaling are therefore necessary to provide insight into the brain processes that are active during navigation in VR environments.

## Conclusion

Otoconia-deficient *tilted* mice and their control littermates performed a reference memory task on small and large versions of the Barnes maze. On both mazes, control mice preferentially used an efficient spatial search strategy by the end of training, whereas *tilted* mice showed this preference on the large maze. The only difference between the mazes was size, suggesting that the greater linear distance between the goal and alternatives on the large maze may have enabled more efficient navigation. Thus, distance or other task demands may determine the degree to which otolith dysfunction affects the accuracy of navigation.

## Data Availability

The raw data supporting the conclusions of this article will be made available by the authors, without undue reservation.
