# Corrigendum: Task demands influence search strategy selection in otoconia-deficient mice

**DOI:** 10.3389/fneur.2025.1621211

**Published:** 2025-05-16

**Authors:** Ryan M. Yoder, Lucas C. Carstensen, Keshav Jagannathan

**Affiliations:** ^1^Department of Psychology, Purdue University Fort Wayne, Fort Wayne, IN, United States; ^2^Department of Mathematics & Statistics, Coastal Carolina University, Conway, SC, United States

**Keywords:** otolith organs, spatial, self-movement, reference memory, Barnes maze

In the published article, there was an error in the Results, Small Barnes maze search strategy analysis, Paragraph 2. One statistical result (chi square value) reported in the text was incorrect.

This sentence previously stated:

“A chi-square test for independence revealed that the frequencies of spatial, serial, and mixed strategies were different between training days 1 and 8 for control mice (χ2 (2, *N* = 64) = 11.22, *p* < 0.01) and tilted mice (χ2 (2, *N* = 64) = 6.68, *p* < 0.05).”

The corrected sentence appears below:

“A chi-square test for independence revealed that the frequencies of spatial, serial, and mixed strategies were different between training days 1 and 8 for control mice [χ^2^ (2, *N* = 64) = 11.22, *p* < 0.01] and *tilted* mice [χ^2^ (2, *N* = 64) = 9.18, *p* < 0.05].”

The authors apologize for this error and state that this does not change the scientific conclusions of the article in any way. The original article has been updated.

